# Prognostic analysis of m6A-related lncRNAs as potential biomarkers in intrahepatic cholangiocarcinom

**DOI:** 10.3389/fgene.2022.982707

**Published:** 2022-09-08

**Authors:** Guodong Shi, Junjie Wang, Weiqi Wang, Min Chen, Xiaoxuan Liu, Yufan Zheng, Yi Fu, Minghua Wang, Xiaojie Zhang

**Affiliations:** ^1^ Department of Biochemistry and Molecular Biology, Medical College, Soochow University, Suzhou, China; ^2^ Department of Human Anatomy, Histology and Embryology, Medical College, Soochow University, Suzhou, China; ^3^ Department of Experimental Center, Medical College, Soochow University, Suzhou, China

**Keywords:** intrahepatic cholangiocarcinoma, N6-methyladenosine, long non-coding RNA, gene co-expression network, prognostic signature

## Abstract

Intrahepatic cholangiocarcinoma (iCCA) patients had no obvious symptoms at early stage and poor postoperative survival. Therefore, the establishment of an iCCA prognostic prediction model to carry out refined management of iCCA patients is expected to improve the survival of the iCCA patient population. In this paper, we analyzed the expression profiling data of patients from 32 iCCA tissues and eight paracancerous tissues in The Cancer Genome Atlas (TCGA) database. Perl software was used to separate M6A-related genes and lncRNAs from expression matrix files obtained from the TCGA database. The differentially expressed lncRNAs in the iCCA samples and the normal samples were screened out by differential analysis using the R package limma, and the m6A-related lncRNAs were further screened by Pearson correlation analysis. WGCNA clustering analysis constructs a random network to extract the module genes most related to iCCA, and take the intersection of differentially expressed lncRNAs related to m6A. Univariate Cox screening was performed for the intersection lncRNAs that had significant influence on the prognosis of iCCA patients, and further screening was performed by LASSO method and multivariate Cox regression analysis. Risk model was constructed and prognostic ability was evaluated according to risk score. In conclusion, we identified four m6A-related lncRNAs with potential prognostic value in iCCA, and established a novel m6A-related lncRNA-based prognostic model, which can be used as an independent prognostic factor to predict the prognosis of clinical patients.

## Introduction

Cholangiocarcinoma (CCA) is a malignant tumor that occurs in the lining epithelium of the bile duct system. It is the most common biliary tract malignant tumor and the second most common primary liver malignant tumor, accounting for about 2% of all cancer patients worldwide. Cholangiocarcinoma can be further divided into intrahepatic Cholangiocarcinoma (iCCA), perihilar Cholangiocarcinoma (pCCA) and distal Cholangiocarcinoma (dCCA), the latter two can be collectively referred to as extrahepatic Cholangiocarcinoma (ECC) (Massarweh and El-Serag, 2017; Rizvi et al., 2018). Relevant studies show that China is one of the Asian countries with a high incidence of intrahepatic cholangiocarcinoma. Due to the insignificant early symptoms of intrahepatic cholangiocarcinoma and the lack of effective screening markers, patients are often diagnosed in the middle and late stages, missing the best opportunity for surgery, the treatment effect is not ideal, and the prognosis of the patient is poor (Shindoh and Vauthey, 2014).

Epigenetics is the regulation of gene expression in a manner independent of genome sequence and plays a very important role in various diseases and tumors (Feinberg, 2018). In general, epigenetic regulation refers to the diverse and reversible chemical modifications of DNA and histones. In addition to DNA and histones, intracellular RNAs (mRNA, lncRNA,snRNA,etc.) also have different types of post-transcriptional modifications, among which the most common is N6-adenosine methylation (m6A) (Roundtree and He, 2016). m6A methylation refers to the addition of a methyl group to the sixth nitrogen atom of adenine to form 6-methyladenine, which is about 0.1–0.4% in total RNA of adenosine is methylated by m6A (Zhang C. Y. et al., 2019). As the most abundant post-transcriptional modification at the RNA level (Meyer et al., 2012), m6A methylation modification is a dynamic, reversible modification process mediated by m6A WER (“ writers”, “Erasers” and “Reader”) (Panneerdoss et al., 2018). Among them, methyltransferases (writers), which are mainly responsible for adding methyl groups to specific sites in the mRNA of specific target genes, are composed of catalytic subunit methyltransferase-like 3 (*METTL3*), methyltransferase-like 14 (*METTL14*), Wilms’ tumor 1-associating protein (*WTAP*) and other proteins (Scholler et al., 2018). Demethylases (erasers), including proteins such as *ALKBH5* and *FTO*, function to recognize and remove m6A methylation (Xu et al., 2020). In addition, *YTHDF* and *YTHDC* family members are generally considered to be m6A methylated readers, which can specifically recognize and bind m6A methylation sites and induce their corresponding functions (Yue et al., 2019). Growing evidence suggests that m6A modifications play important biological functions in mammals (Kato and Natarajan, 2019; Mayo et al., 2021). Long non-coding RNA (lncRNA) is a non-coding RNA with a length of more than 200 nucleotides, which generally does not encode proteins and polypeptides. Compared with mRNA, it has stronger spatiotemporal specificity and lower conservation between species (Rinn et al., 2007; Peng W. X. et al., 2017). LncRNAs can participate in the regulation of genomic imprinting, stem cell pluripotent differentiation, embryonic development, cardiac development, hematopoiesis and immune systems, and endocrine systems and other biological physiological processes (Bhan et al., 2017; Huang, 2018). And lncRNA can act as molecular scaffolds in the nucleus, assist alternative splicing, regulate chromosome structure, regulate translation in the cytoplasm, promote or inhibit mRNA degradation, adsorb miRNA, etc (Satpathy and Chang, 2015).

M6A modification affect the function of lncRNAs through multiple regulatory mechanisms. On the one hand, m6A modification acts on the RNA-DNA triple helix structure and regulates the relationship between lncRNA and specific DNA sites. On the other hand, m6A modification provides binding sites for methylated readers (readers) or regulates the structure of local RNAs, thereby inducing the binding of RNA-binding proteins (RBPs) and regulating the function of lncRNAs(Huang et al., 2020).

Weighted gene co-expression network analysis (WGCNA) is a comprehensive analysis technology based on biological network, which can identify a class of genes (or proteins) that are co-expressed, associate clustering modules with phenotypes through algorithms, and explore the core of the modules gene (or protein) (Langfelder and Horvath, 2008). The protein interaction network is composed of interactions between proteins, and participates in biological processes such as signal transmission, gene expression regulation, and material metabolism. It can be used for polygenic analysis of complex mechanisms and large-scale datasets, and can also be used to reveal associations between genes in different samples (Zhang et al., 2021).

The LncSEA database integrates the collection of lncRNA functional information from more than 20 published databases, including lncRNA functional collections including downstream regulation, as well as TF ChIP-seq, DNase-seq, ATAC-seq, and H3K27ac ChIP-seq. This database focuses on the published information of various human lncRNAs, and can perform annotation and enrichment analysis on the lncRNA collections submitted by users, providing more than 40,000 reference lncRNA collections, including 18 types and 66 subtypes, including more than 50,000 lncRNAs(Chen et al., 2021).

The Cox proportional hazards model is essentially a regression model commonly used in statistics in medical research to study the association between a patient’s survival time and one or more predictor variables. It is suitable for both quantitative predictors and categorical variables (Cox, 1972). Univariate Cox analysis is usually used to remove collinearity, but it may lead to synergistic effects brought by other variables, so multivariate Cox regression is performed to correct other factors, which is often used for survival analysis data modeling, etc. Thus, an m6A-related prognostic model may be helpful in the understanding and management of iCCA (Huang and Liu, 2006; Li B. et al., 2016). Here, we investigated the prognostic and immunologic significance of m6Arelated lncRNAs and developed an m6A-related lncRNA prognostic model to predict survival outcomes in patients with iCCA.

## Materials and methods

### Data sources

Using The Cancer Genome Atlas (TCGA-GDC, Data release 32.0)which was updated on 29 March2022, we obtained transcriptome-sequencing (RNA-seq) information from 32 iCCA samples and eight adjacent non-tumor samples with corresponding clinical data. Perl (Strawberry Perl v5.30.2) was used to extract the gene expression information of lncRNAs, and Python (Python 3.6) was used to match 23 m6A-related genes (*METTL3, METTL14, METTL16, WTAPI, VIRMA, ZC3H13, RBM15, RBM15B, YTHDC1, YTHDC2, YTHDF1, YTHD F2, YTHDF3, HNRNPC, FMR1, LRPPRC, HNRNPA2B1, IGFBP1, IGFBP2, IGFBP3, RBMX, FTO, ALKBH5*) expression matrix (Deng et al., 2018; Chen et al., 2019).

### Differential lncRNAs analysis and m6A-related lncRNAs identification

Using the limma R package, the sorted lncRNAs expression matrix was used for gene difference analysis (|log2FC|>1, p< 0.05), and these up-regulated or down-regulated genes were plotted by plotting volcano plot and heatmaps shows up. Pearson correlation analysis was used to identify m6A-related lncRNAs(|Pearson R| > 0.35, *p* < 0.025) (Xu et al., 2021).

### Construction of a weighted gene functional co-expression network

The gene co-expression network analysis was constructed using the WGCNA package of the R. In order to ensure that it conforms to the scale-free network distribution, the “pickSoftTreshold” function in the WGCNA package calculates the correlation coefficient of the β value and the mean value of gene connectivity, and selects an appropriate soft threshold β to make the network more flexible. Then, a one-step method is used to construct the related gene network, the adjacency matrix is converted into a topological overlap matrix TOM, and a hierarchical clustering tree of a gene is generated by hierarchical clustering. Calculate gene significance (GS) and module significance (MS) to measure the significance of genes and clinical traits, and analyze significant associations between modules and models.

### Network module establishment and functional annotation of lncRNAs

The most significant associations with cancer traits in the WGCNA modules were selected for study and compared with the association results using Python. Retaining the part of the most significant modules of WGCNA that coincides with the correlation results, and finally obtaining the correlation network of m6A-related lncRNAs. Use Gephi (v0.9.2) software to draw the network, export the network nodes, modules and in- and out-degree information, and import the lncRNAs with higher weights into the lncSEA (http://bio.liclab.net/LncSEA/index.php) for Annotation Analysis.

### Prognostic analysis

Univariate Cox regression analysis was performed for all lncRNAs in the network (KM < 0.04, *p* < 0.05), and then the LASSO regression method was used to further screen m6A-related prognostic lncRNAs. Perform multivariate Cox regression analysis for prognostic modeling and draw forest plots. Using the Kaplan-Meier method of the “survival” package to perform survival analysis on the screened genes, and draw survival curves. According to the median prognostic risk value, the samples of patients with intrahepatic cholangiocarcinoma were divided into low-risk group and high-risk group, and the risk level between the low-risk group and the high-risk group, as well as the survival status and survival time of the patients were drawn as a scatterplot, and then use the Kaplan-Meier method to draw survival curves for the risk model. Compare risk values to various clinical traits and perform independent prognostic analyses to test the prognostic power of risk models. The Roc curve was drawn to represent the accuracy of predicting patient survival through the model, and the accuracy was judged by the area under the curve.

### Statistical analysis

In this study, the R (version 4.2.0) and RStudio software were utilized to carry out the statistical analysis and figure preparation. The quantitative data from the control and the experimental groups were compared by using the student t test. Pearson correlation was used to calculate the correlation coefficients. *p*-values less than 0.05 were defined as statistically significant.

## Results

### Screening of differentially expressed and m6A-related lncRNAs in iCCA

**FIGURE 1:**
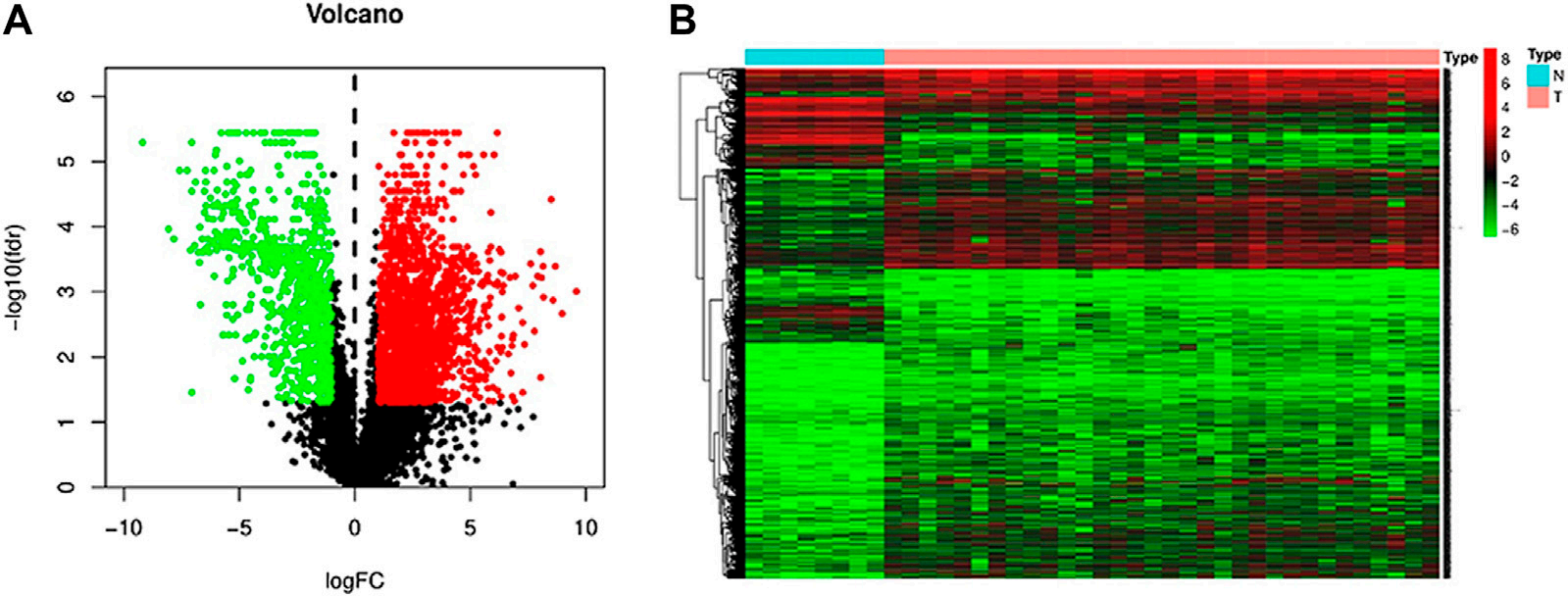
Differentially expressed lncRNAs in iCCA patients and adjacent normal patients. **(A)** Volcano map of differentially expressed lncRNAs (red dots represent up-regulated genes, green dots represent down-regulated genes). **(B)** Cluster heat map of hetero-expressed gene.

In order to screen and identify m6A-related lncRNAs, we downloaded the transcriptome and clinical data of iCCA samples and adjacent normal samples from the TCGA database (matched to the expression matrix containing 22 m6A regulators and lncRNAs). The limma was used to analyze the differentially expressed lncRNAs in normal and iCCA samples, and finally 3,099 differential lncRNAs were obtained (|log_2_ FC|>1, p< 0.05). Differentially expressed genes are plotted on a volcano plot (Figure 1A), where red represents up-regulated genes and green represents down-regulated genes. At the same time, a heatmap (Figure 1B) is drawn, where red represents gene up-regulation and green represents gene down-regulation. Pearson correlation analysis was used to identify 2,848 lncRNAs associated with m6A (|Pearson R|>0.35, p< 0.025).

### Construction of weighted gene co-expression network analysis

**FIGURE 2:**
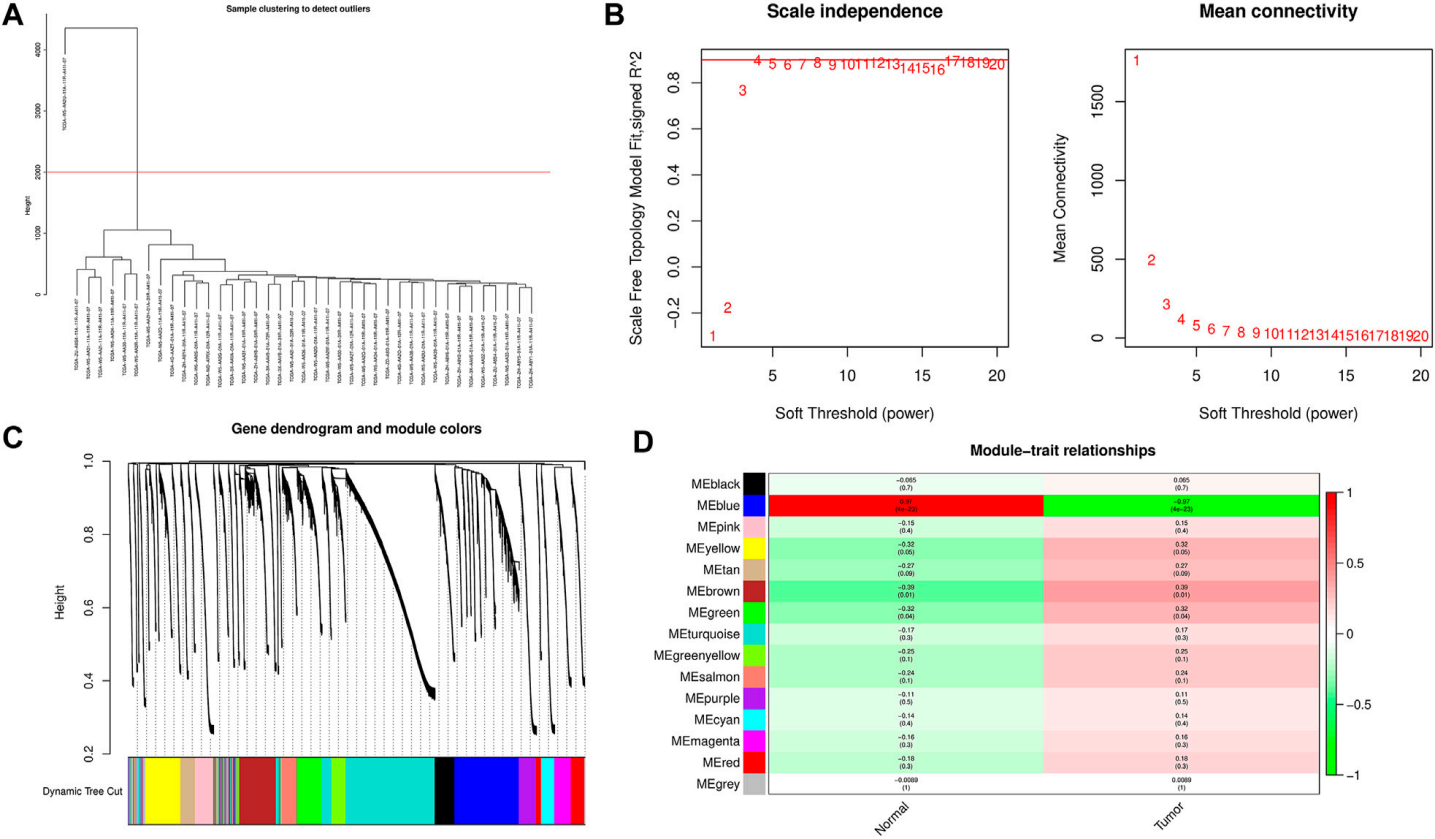
Construction of co-expression modules related to iCCA. **(A)** Set the height to 2000 to remove outliers. **(B)** Determine the best soft threshold (In the process of module selection, the adjacency matrix is converted into a topology matrix, and the optimal soft threshold β = 4 is determined). **(C)** Cluster tree of coexpressed gene modules **(D)** The correlation between the gene module and clinical information (The redder the color, the higher the correlation; the figure in the figure is the Pearson correlation coefficient, in brackets. The number is the corresponding *p* value).

WGCNA analysis was performed using m6A regulators and expression matrix of lncRNAs. After setting the height to 2000, one outlier sample was removed, and 32 iCCA samples and seven normal samples were retained for subsequent analysis (Figure 2A).The pick Soft Threshold function is used to weight the parameter β. The results show that when *β* = 4, the scale-free topology fitting index R2 reaches 0.9. At this time, the average network connection corresponding to this threshold is close to the scale-free network (Figure 2B). Obtain the dissimilarity matrix dissTOM between genes, perform hierarchical clustering on the dissimilarity matrix to obtain a gene clustering tree, and combine high similarity modules to obtain 15 modules, that is, 22 m6A regulators and 13,975 lncRNAs are finally divided into 15 co-expression network modules, successfully constructed a scale-free network and completed the division of gene modules (Figure 2C). According to the heat map results of the correlation between modules and traits in WGCNA, we selected the blue module with the highest correlation coefficient (r = 0.97, p< 0.01) for follow-up research (Figure 2D).

### Network module establishment and functional annotation of lncRNAs

**FIGURE 3:**
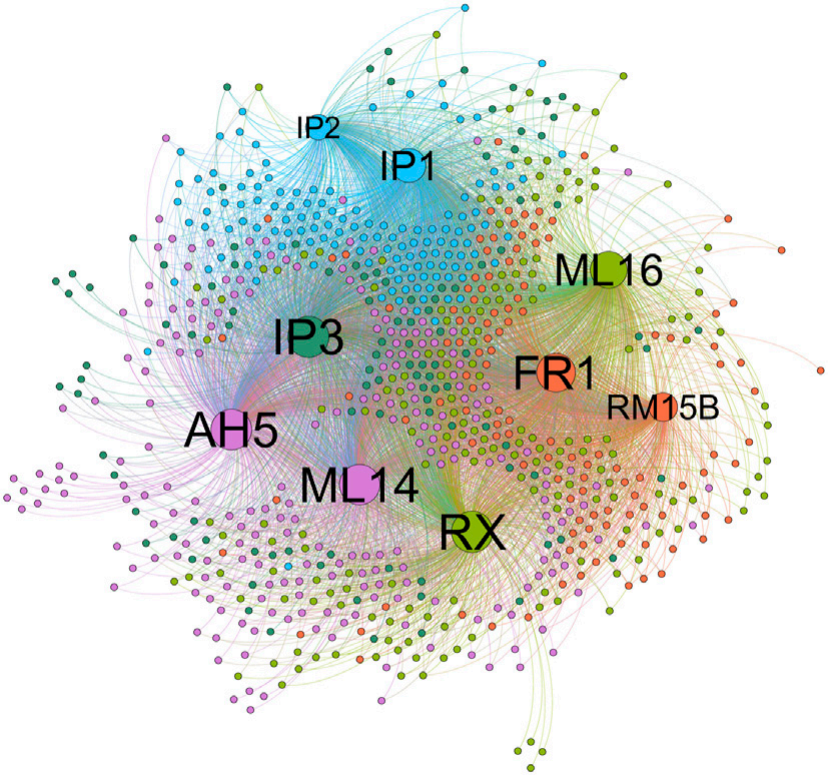
Correlation coexpression network graph (Use Gephi software’s Force Atlas layout to map the network).

**FIGURE 4:**
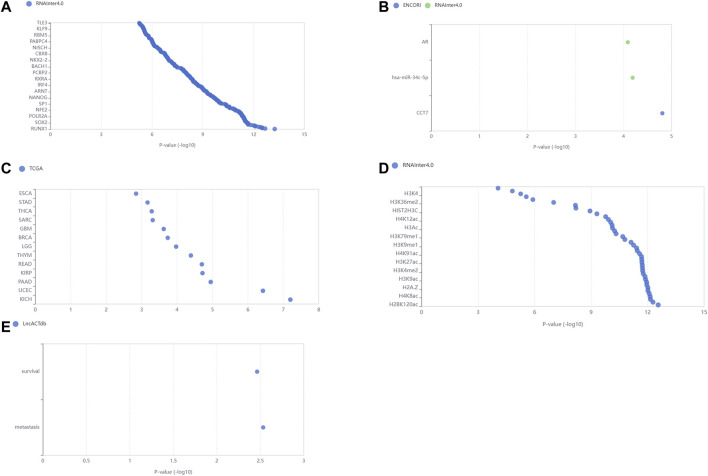
LncSEA platform annotation analysis results. These lncRNAs are closely associated with **(A)** RNA Protein interaction; **(B)** RNA interaction; **(C)** survival; **(D)** RNA Histone modification; **(E)** Cancer functional state.

**FIGURE 5:**
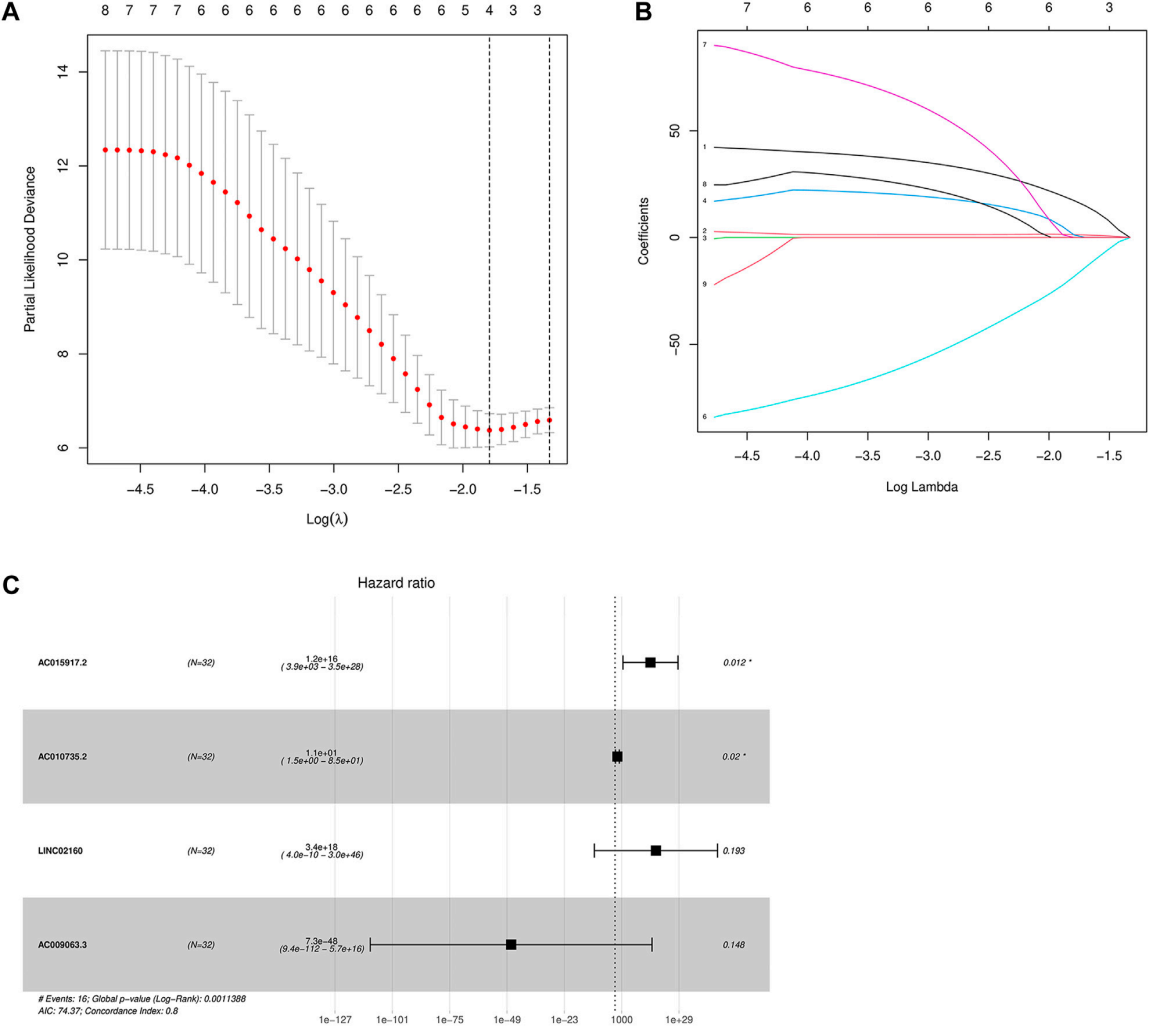
**(A,B):** LASSO Cox regression analysis determined four m6A-related lncRNAs; **(C)** Multivariate Cox forest diagram.

Comparing the node-node information in the blue module with the node-node information of the correlation, and taking the intersection, a total of 723 nodes are obtained, corresponding to 1,560 edges, that is, the correlation co-expression network. By mapping the network using the Force Atlas layout of Gephi software, the network was divided into five modules, and the labels of the m6A regulators were abbreviated and displayed, resulting in nine m6A regulators in the WGCNA highly correlated modules (Figure 3). The network map of Gephi is derived, and the information of m6A regulators is selected (Table 1). The co-expression of these nine m6A regulators regulates a large number of lncRNAs. 207 lncRNAs with a weight of nine were imported into the LncSEA platform for annotation, and a total of five results were obtained, namely 1) RNA Protein interaction (Figure 4A); 2) RNA interaction (Figure 4B); 3) survival (Figure 4C); 4) RNA Histone modification (Figure 4D); 5) Cancer functional state (Figure 4E).

**TABLE 1:** Nine m6A regulators information tables in the WGCNA high correlation module.

ID	Label	Modularity_class	Degree
RBMX	RX	4	616
IGFBP3	IP3	3	612
ALKBH5	AH5	0	606
METTL14	ML14	0	599
FMR1	FR1	1	569
METTL16	ML16	4	534
IGFBP1	IP1	2	484
RBM15B	RM15B	1	390
IGFBP2	IP2	2	331

### Cox analysis and risk model establishment

The lncRNAs related to the above nine m6A regulators were screened by the single-factor Cox method (Table2), and the four m6A-related lncRNAs related to prognosis were screened by the LASSO method (Figure 5A). On this basis, further multivariate Cox regression analysis was performed, and it was found that four lncRNA, *AC015917.2*, *AC010735.2*, *LINC02160*, and *AC009063.3*, may have a significant impact on the prognosis of patients with iCCA (Figure 5B). Construction of a multivariate Cox model of prognostic risk in patients with iCCA using four lncRNAs (Table 3). According to the multivariate COX regression analysis, the regression coefficient of each lncRNA was calculated as the hazard ratio (HR), and the risk score formula was obtained as the prognostic model:

Risk Score=(AC015917.2×37.0)+(AC010735.2×2.4)+(INC02160×42.7)+[C009063.3×(−108.5)]

**TABLE 2:** The univariate Cox regression analysis demonstrating nine m6A-related lncRNAs.

Gene	HR	*p* Value
AC015917.2	4.6976E+14	0.008212199
AC010735.2	13.47635223	0.004377585
AL353693.1	161625.4708	0.007849688
LINC02160	2.44E+24	0.045667139
AC008708.1	175,502,748	0.008706877
AC009063.3	7.26E-67	0.040178505
AC026316.3	1.08E+37	0.038001379
AC006504.2	7.98004E+19	0.016435749
AP001172.1	1.02726E+15	0.02532585

**TABLE 3:** Multivariate COX modeling results.

Gene	Coef	Risk score	*p* Value
AC015917.2	36.99335531	1.16415E+16	0.01163183
AC010735.2	2.413316583	11.17094916	0.019585653
LINC02160	42.6833156	3.44452E+18	0.193405813
AC009063.3	−108.5397043	7.27E-48	0.148162053

Draw a forest diagram (Figure 5C). According to the risk model, the risk value of all patients with intrahepatic cholangiocarcinoma can be obtained.

### Survival analysis and independent prognostic analysis

**FIGURE 6:**
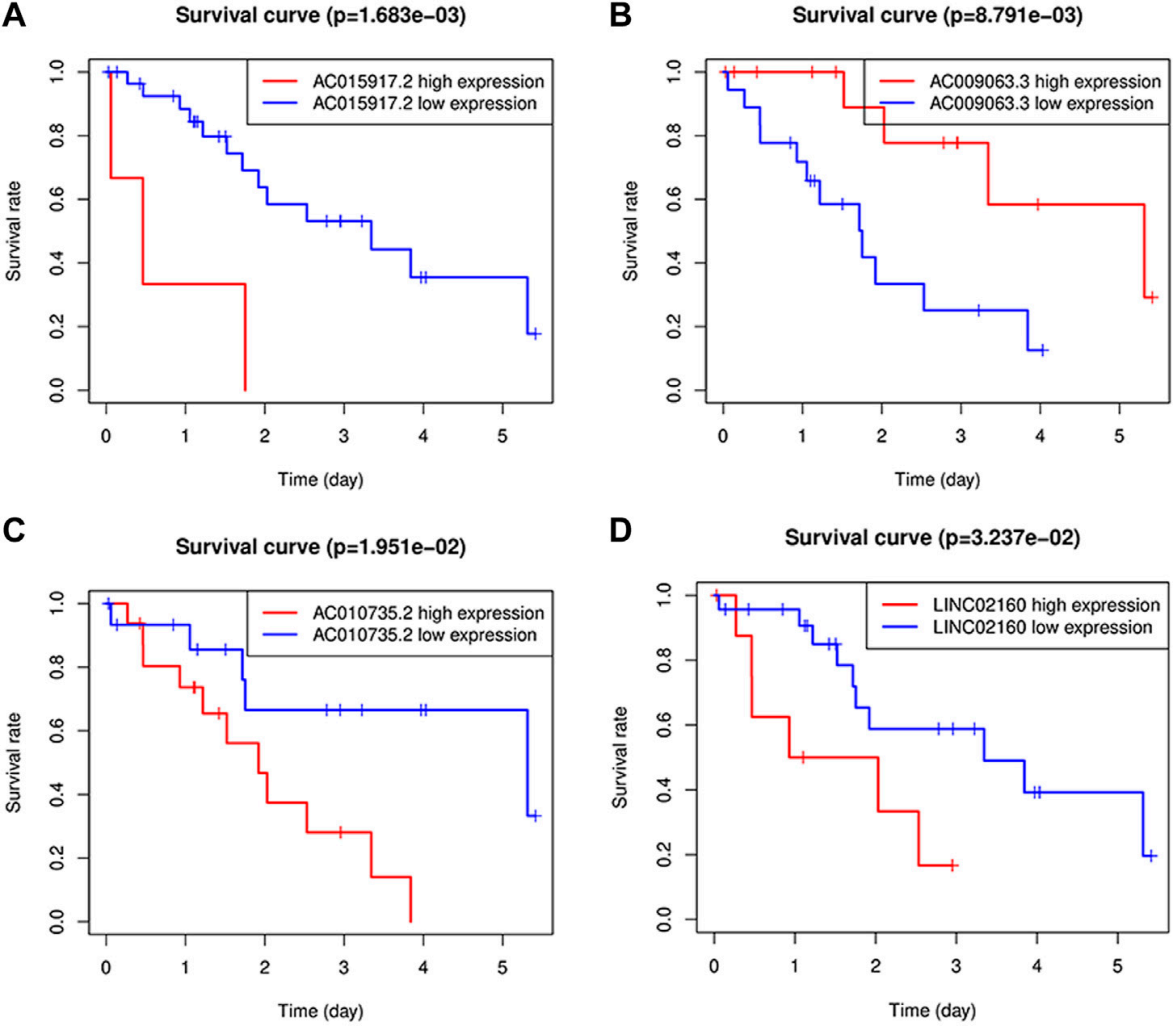
Kaplan-Meier survival analysis of four lncRNAs; **(A-D)**: Survival curve, where AC015917.2 AC010735.2, LINC02160 high expression, AC009063.3 patients with low expression of iCCA OS is poor.

**FIGURE 7:**
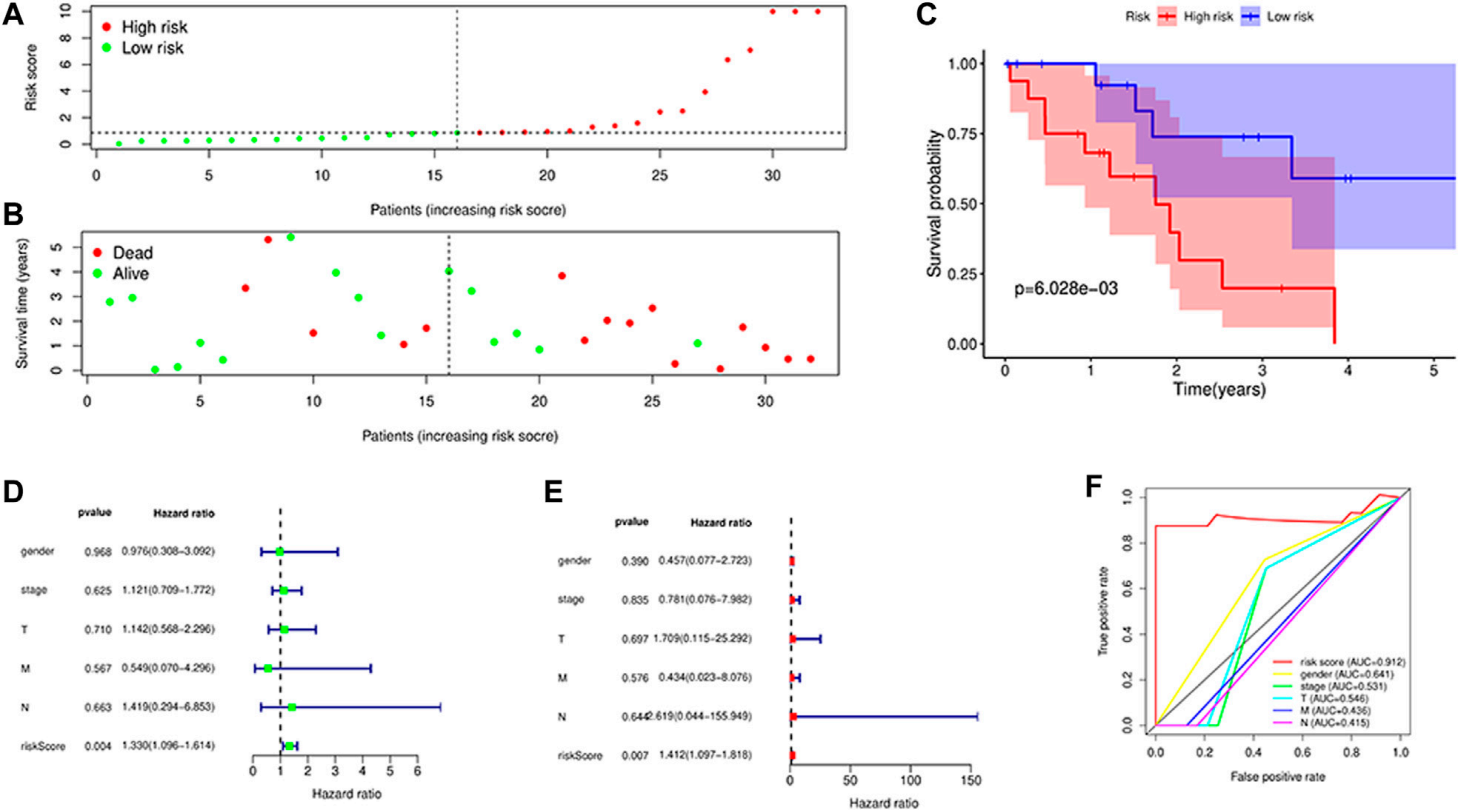
Survival analysis. **(A,B)**: Distributions of risk scores and survival status, Survival was longer in the low-risk group than in the high-risk group; **(C)**: Kaplan–Meier curves for the High risk and low risk patients. The survival curve showed low survival in the high-risk group; **(D,E)**: Univariate **(D)** and multivariate **(E)** Cox regression analysis in the simultaneously demonstrated the independent prognostic value of the risk score; **(F)**: ROC curves for predicting risk score, gender,stage,T, M, N stage.

The four long-chain non-coding expression matrices obtained in the model were combined with the survival time and survival status of the clinical data, and the survival curve was drawn (Figure 6(A-D)). It was found that *p* = 0.0017 for *AC015917.2*, *p* = 0.0195, *p* = 0.0324 for *LINC02160*, and *p* = 0.0088 for *AC009063.3*, indicating that these four lncRNAs may have an impact on the prognosis of iCCA patients. The median of the patient’s risk value was used as the threshold, and the patients were divided into high-risk group and low-risk group (Figure 7A). Status and survival time were analyzed. (Figure 7B). After dividing the samples into high and low risk groups according to the median prognostic risk level, the survival analysis result *p* = 0.006 (Figure 7C). It showed that the OS of the low-risk group was longer than that of the high-risk group, which preliminarily verified the prognostic ability of this model. Univariate and multivariate independent prognostic analyses were performed on the above four m6A-related lncRNAs respectively (Figures 7D, E), indicating that the risk model of the four m6A-related lncRNAs was independent of other clinicopathological parameters (such as gender, stage, TNM stage). The Roc curve showed the risk model AUC = 0.912, which suggests that the model is far superior to other clinical traits in predicting patient surviva (Figure 7F).

## Discussion

Cholangiocarcinoma is a common primary liver malignancy worldwide, and its incidence is increasing year by year, seriously endangering the life and health of patients (Florio et al., 2020). Because its early pathogenesis is complex and difficult to detect, patients are often in the middle and late stages once diagnosed, and the prognosis of patients is poor. As one of the common subtypes of cholangiocarcinoma, iCCA also has the characteristics of high metastasis and high invasion (Moeini et al., 2016). Therefore, further search for biological prognostic markers of iCCA is conducive to further understanding the pathogenesis of iCCA and providing new ideas for the development of new treatment methods.

With the in-depth study of lncRNAs, we have gained new understanding of cancer. In cancer, abnormal expression of lncRNAs determines disease progression, explains patient resistance, and can predict patient prognosis (Bester et al., 2018; Zhang W. et al., 2019). Current evidence suggests that *H19* is overexpressed in prostate cancer, glioblastoma, and breast cancer and is associated with cancer progression (Bauderlique-Le Roy et al., 2015; Jiang et al., 2016; Peng F. et al., 2017). High expression of *HIF2PUT* is a marker of aggressive osteosarcoma, and overexpression of *HIF2PUT* is associated with an extremely poor prognosis in patients (Li W. et al., 2016). At the same time, more and more evidences indicate that m6A is related to tumor proliferation, differentiation, invasion and metastasis (Cui et al., 2017; Kwok et al., 2017). And it has been deeply studied in a variety of solid tumors (Chen et al., 2019). Therefore, the study of m6A related lncRNA is of great significance for the occurrence, development and treatment of iCCA.

In this study, the gene expression map data of iCCA in the TCGA database was used to separate the mRNA and lncRNAs in the gene matrix file by using Perl software, and the lncRNAs gene expression matrix file of iCCA was obtained, and aggregated by WGCNA. Class analysis to further screen out differentially expressed lncRNAs in normal and iCCA patients. After correlation analysis of m6A-related genes, lncRNAs with differential expression in m6A-related genes were further obtained. By drawing a Venn diagram to take the intersection of the two, taking the lncRNAs with higher weights in the network, and analyzing them in the LncSEA database, it is found that the overexpression of lncRNAs co-expressed with m6A is likely to cause the deterioration of cholangiocarcinoma. The above lncRNAs were screened by univariate Cox regression, LASSO method screening and multivariate Cox regression method, and obtained four lncRNAs related to the prognosis of patients with intrahepatic cholangiocarcinoma, namely *AC015917.2*, *AC010735.2*, *LINC02160, AC009063.3*. Based on this, a prognostic model was established, and then the prognosis was predicted according to the risk score. The risk score calculated by these four m6A-related lncRNAs as risk signals can relatively accurately predict the prognosis of patients with intrahepatic cholangiocarcinoma, and is an independent prognosis factor for patients with iCCA.

In conclusion, we identified four m6A-related lncRNAs with potential prognostic value in iCCA and developed a prognostic. They may be applied in the determination of the treatment efficacy in patients with iCCA. However, this study also has certain limitations. The data analyzed are all from the TCGA database, and the data analysis results may be biased. A large amount of relevant data information of iCCA patients is still needed to verify the predictive power of this prognostic model. Therefore, in the follow-up study, we will continue to collect the relevant data of iCCA patients and their survival information, and combine the conclusions of this paper to conduct further clinical validation of the screened related genes and the constructed prognostic model.

## Data Availability

Publicly available datasets were analyzed in this study. This data can be found here: https://portal.gdc.cancer.gov/repository.
